# Clinical Characteristics and Cytokines of Children With Lower Respiratory Tract Infection Caused by Influenza A After COVID‐19

**DOI:** 10.1155/cjid/5007103

**Published:** 2026-01-23

**Authors:** Yue Qi, Xinqian Xu, Hongrong Dan, Miao He, Qinxin Zheng, Xing Liu, Li Xiang, Yingshun Zhou, Cheng Li

**Affiliations:** ^1^ Department of Pediatrics, The Affiliated Hospital, Southwest Medical University, Luzhou, China, swmu.edu.cn; ^2^ Sichuan Clinical Research Center for Birth Defects, Sichuan, China; ^3^ Department of Cardiology, The Affiliated Hospital, Southwest Medical University, Luzhou, China, swmu.edu.cn; ^4^ Collaborative Innovation Center for Prevention and Treatment of Cardiovascular Disease of Sichuan Province, Southwest Medical University, Luzhou, China, swmu.edu.cn; ^5^ Department of Pathogen Biology, The Public Platform of the Pathogen Biology Technology, School of Basic Medicine, Southwest Medical University, Luzhou, China, swmu.edu.cn

**Keywords:** children, clinical characteristic, COVID-19, cytokine, influenza A

## Abstract

**Background:**

To date, there is a lack of research on the clinical characteristics and cytokine profiles in children with influenza A following COVID‐19 infection.

**Methods:**

Children with influenza A lower respiratory tract infection who were hospitalized in the Department of Pediatrics of the Affiliated Hospital of Southwest Medical University from March 15 to April 15, 2023, were divided into mild and severe groups according to their symptoms, followed up on the COVID‐19 infection status by telephone, and selected those who had been infected with COVID‐19 for comparison of clinical characteristics and cytokines.

**Results:**

An analysis of influenza A case in children with prior SARS‐CoV‐2 infection revealed that the proportion of previous infection was significantly higher in the severe group compared to the mild group. Univariate analysis indicated significant differences between the two groups in the incidence of convulsions, concurrent or secondary bacterial infections, white blood cell (WBC) count, lymphocyte percentage (L%), neutrophil percentage (N%), and interferon‐induced protein‐10 (IP‐10) (all *P* < 0.05). Binary logistic regression and receiver operating characteristic (ROC) curve analysis identified IP‐10 and WBC as independent risk factors for severe influenza A following SARS‐CoV‐2 infection, the combination of IP‐10 and WBC demonstrated high diagnostic accuracy for disease severity.

**Conclusion:**

IP‐10 and WBC count serve as diagnostic biomarkers for severe influenza infection following SARS‐CoV‐2 infection, and their combination significantly enhances diagnostic performance.

## 1. Introduction

Influenza, a highly contagious respiratory disease, is prevalent worldwide [1]. The most recent pandemic caused by the H1N1 influenza virus occurred in 2009 and affected approximately 60.8 million individuals globally [2]. According to available data, an estimated 20%–30% of children worldwide are affected by influenza annually, resulting in approximately 28,000 deaths among those under 18 years of age due to influenza‐associated lower respiratory tract infections (LRTIs). The majority of these fatalities occur in children under the age of 4 [3]. Compared to adults, children have an immature immune system, making them more susceptible to influenza infection, which frequently involves the lower respiratory tract and may lead to pneumonia and other severe complications [4]. Influenza A is the most common type of influenza virus and the only one known to have the potential to cause pandemics [5].

Coronavirus disease 2019 (COVID‐19), caused by severe acute respiratory syndrome coronavirus 2 (SARS‐CoV‐2), was first identified in Wuhan, China, in December 2019. Since then, it has rapidly spread to numerous countries worldwide, imposing a substantial burden on global public health, economic systems, and social stability. Like influenza viruses, SARS‐CoV‐2 exhibits high transmissibility and genetic variability [6]. Following the implementation of stringent non‐pharmaceutical interventions (NPIs) in China, the incidence of SARS‐CoV‐2 infection among children has been significantly lower than that in adults, particularly the elderly population. Although most infected children remain asymptomatic or exhibit mild symptoms, a subset may develop severe complications, including progression to pneumonia with acute respiratory distress syndrome (ARDS), encephalitis, and multi‐organ dysfunction—clinical manifestations comparable to those observed in severe influenza A infections [7, 8].

Evidence indicates that critically ill children infected with either virus commonly exhibit pathological changes such as capillary epithelial barrier damage, inflammatory cell infiltration, alveolar wall congestion and edema, and hypoxia. These pathological alterations and disease progression are driven not only by the intrinsic pathogenicity of the virus but also by excessive immune responses, which represent a key mechanism contributing to the exacerbation of lung injury [9–11].

Cytokines serve as critical mediators of the innate antiviral immune response and play a pivotal role in maintaining the balance between cellular and humoral immunity [12, 13]. Previous studies on cytokine profiles in influenza and SARS‐CoV‐2 infections have demonstrated that immune dysregulation, along with elevated levels of chemokines (e.g., IP‐10) and proinflammatory cytokines (e.g., IL‐6, TNF‐α), is closely associated with disease progression and severity in viral infections. Yu Xue et al. analyzed sputum samples from patients with influenza A virus pneumonia and found significantly higher levels of IL‐6, IL‐2, and IFN‐γ in severe cases compared to mild ones [14]. In a study of eight Chinese children with SARS‐CoV‐2 infection, researchers reported a marked increase in circulating IL‐1β levels during the early phase of illness [15]. Additionally, a literature review on inflammatory cytokine profiles in Egyptian children and adolescents revealed that elevated serum IP‐10 levels independently predicted severe COVID‐19 pneumonia [16]. However, these studies focused exclusively on single‐virus infections and were conducted prior to the implementation of China’s “dynamic zero‐COVID” policy, highlighting a critical gap in understanding the clinical characteristics and inflammatory cytokine dynamics of post‐SARS‐CoV‐2 influenza A infections.

By systematically reviewing existing literature on pediatric infections with influenza A and SARS‐CoV‐2, this study compares the clinical features, laboratory findings, and inflammatory cytokine profiles between mild and severe cases of influenza A‐induced LRTIs following SARS‐CoV‐2 infection. The objectives are to evaluate the diagnostic value of inflammatory cytokines in assessing disease severity after SARS‐CoV‐2 infection, identify potential shifts in the clinical presentation of influenza A before and after the pandemic, and compare these manifestations with those of SARS‐CoV‐2 monoinfection.

## 2. Methods

### 2.1. Population

**Figure 1:**
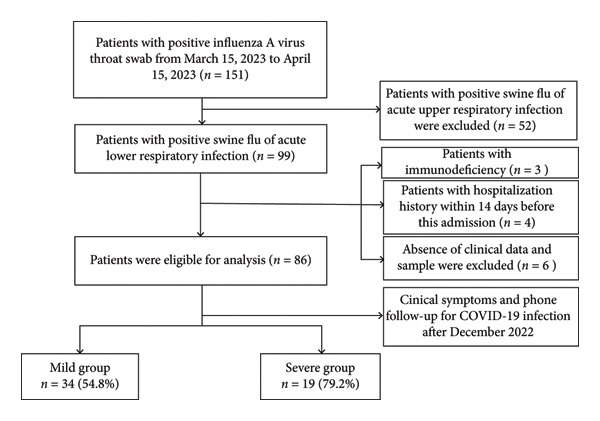
Flow chart of the study design and enrollment.

This study conducted a retrospective cohort analysis of children aged 28 days to 14 years who were hospitalized in the Department of Pediatrics at the Affiliated Hospital of Southwest Medical University between March 15, 2023, and April 15, 2023, due to lower respiratory tract pneumonia caused by influenza A virus infection. Patient data were extracted from the Hospital Information System (HIS). Inclusion criteria required fulfillment of the diagnostic criteria for acute LRTI, defined as acute tracheobronchitis or pneumonia caused by any pathogen, accompanied by influenza‐like symptoms and confirmed by a positive influenza A‐specific throat swab test [17]. Exclusion criteria included: (1) presence of primary or secondary immunodeficiency disorders; (2) hospitalization duration less than 24 h; and (3) prior hospitalization within 14 days before the current admission. Patients meeting the inclusion criteria and exhibiting one or more of the following complications—ARDS, acute respiratory failure, secondary bacterial pneumonia, sepsis, acute myocarditis, acute pericarditis, acute encephalitis, acute disseminated encephalomyelitis, transverse myelitis, aseptic meningitis, Guillain–Barré syndrome, myositis, or rhabdomyolysis—were classified as having severe influenza A infection. Notably, respiratory distress and convulsions were considered key clinical indicators of disease severity in pediatric patients [18, 19]; all other cases were categorized as mild infections. The history of SARS‐CoV‐2 infection during the period from November 2022 to February 2023 was collected via telephone interviews. Children with no documented prior infection or unclear infection status were excluded. Only those with confirmed previous SARS‐CoV‐2 infection were included in the comparative analysis. The study flowchart is presented in Figure 1.

### 2.2. Collection of Serum

Within 24 h of admission, residual serum samples obtained after routine clinical laboratory testing at the Affiliated Hospital of Southwest Medical University were collected and stored at −80 °C for subsequent experimental use.

### 2.3. Cytokine Assay

In children with LRTIs caused by influenza A virus who had a prior positive diagnosis of COVID‐19, serum concentrations of three cytokines—IP‐10/CXCL‐10, IL‐6, and TNF‐α—were measured using enzyme‐linked immunosorbent assay (ELISA). The detection sensitivities were as follows: IP‐10, 1.0 pg/mL; TNF‐α, 0.1 pg/mL; and IL‐6, 0.1 pg/mL.

### 2.4. Statistical Analysis

Statistical analyses were performed using IBM SPSS Statistics, Version 17 for Windows. For quantitative data, continuous variables with normal distributions were represented by mean ± standard deviation (*x* ± s), and the *T*‐test was used to compare the two groups. The non‐normally distributed continuous variables were expressed as medians (P25, P75) and compared using the Mann–Whitney *U* test. Classification data were expressed as *n* (%) and compared using chi‐square tests. Logistic regression analysis was used to examine the risk factors. Receiver operating characteristic (ROC) curve analysis was employed to assess the diagnostic performance of clinical indicators, with the area under the curve (AUC) serving as the primary metric for evaluation and interpret it as 0.5 < AUC ≤ 0.7 (low diagnostic value), 0.7 < AUC ≤ 0.9 (median diagnostic value), and 0.9 < AUC < 1 (high diagnostic value). *P* < 0.05 was considered statistically significant.

## 3. Results

### 3.1. Children With a History of SARS‐CoV‐2 Infection May Be at Increased Risk of Developing Severe Illness Following Influenza Infection

Among the 151 children enrolled in the study, after excluding those with upper respiratory tract infections, those who did not meet the inclusion criteria, and those with incomplete clinical data, a total of 86 children were identified as having acute LRTIs caused by the influenza A virus. Based on disease severity classification criteria, 62 children were assigned to the mild group and 24 to the severe group. A telephone follow‐up was conducted to collect information on prior SARS‐CoV‐2 infection history. Of these 86 children, 53 had previously been infected with SARS‐CoV‐2 before influenza A virus infection, including 34 (54.8%) in the mild group and 19 (79.2%) in the severe group. Statistical analysis revealed that the proportion of prior SARS‐CoV‐2 infection was significantly higher in the severe group than in the mild group, with a statistically significant difference between the two groups (*p* < 0.05, Table 1).

**Table 1:** Analysis of COVID‐19 infection in children with positive influenza A.

LRTI^a^	Mild group (*n* = 62)	Severe group (*n* = 24)	*p* Value
Positive	34 (54.8%)	19 (79.2%)	< 0.05

^a^Lower respiratory tract infection.

### 3.2. Compared With the Mild Group, WBC and N% in the Severe Group Increased, While L% Decreased

Through statistical analysis of clinical characteristics, we can see there was no statistical difference between the mild and severe groups in gender and age, but the proportion of male children in the two groups was more than girls and the age group was concentrated in 3–5 years old (*p* > 0.05).

Respiratory symptoms were the primary clinical presentation observed in all the children, including fever, cough, nasal obstruction/runny nose, phlegm in the throat, and so on. A few children had other systemic symptoms such as vomiting, diarrhea, convulsions, and so on. There were more children with upper respiratory tract infection symptoms in the mild group, while dyspnea and convulsion symptoms appeared in the severe group. Among them, there was a significant statistical difference in convulsive symptoms between the two groups (*p* < 0.05).

Then the laboratory test results were analyzed, and it can be concluded that WBC and N% in the severe group were higher than those in the mild group, while the L% in the severe group was lower than that in the mild group, and the three indicators were statistically different between the two groups (*p* < 0.05). There were no significant differences in the levels of procalcitonin (PCT), serumamyloid A (SAA), C‐reactive protein (CRP), alanine aminotransferase (ALT), aspartate aminotransferase (AST), Creatinine, urea and human hypersensitive troponin (hs‐TNT) levels between the two groups (*P* > 0.05). There was no statistical difference between the two groups in co‐infection with other viruses (*P* > 0.05), but there was a significant difference in the co‐infection of bacterial infection between the two groups (*P* < 0.05). All detailed results are shown in (Table 2).

**Table 2:** Analysis of clinical characteristics between the mild and severe groups in post‐COVID‐19 influenza A lower respiratory tract infection.

Variables	Mild (*n* = 34)	Severe (*n* = 19)	*p* value
Age (month)	47 (19.75, 80.00)	46 (36.00, 72.00)	0.34
Gender	20 (58.8%)	13 (68.4%)	0.49
Febrile days	4 (2.00, 5.00)	3 (2.00, 4.00)	0.72
Tmax (°C)^a^	39.50 (39.30, 40.05)	39.50 (39.00, 39.80)	0.23
Length of stay in hospital	6 (5.00, 7.00)	7 (4.00, 9.00)	0.17
Underlying disease	6 (17.6%)	6 (31.6%)	0.31
Neurological disease	1 (25%)	3 (75%)	0.13
Congenital heart disease	1 (100%)	0 (0%)	1
Chronic respiratory disease	1 (33.3%)	2 (66.7%)	0.29
Hematological system disease	0 (0%)	1 (100%)	0.36
Chronic digestive disease	1 (100%)	0 (0%)	1
Other diseases	2 (100%)	0 (0%)	0.53
Clinical symptom			
Fever	34 (100%)	17 (89.5%)	0.12
Cough	33 (97.1%)	16 (84.2%)	0.13
Runny nose	13 (38.2%)	6 (31.6%)	0.63
Stridor	6 (17.6%)	2 (10.5%)	0.7
Phlegm rings	24 (70.6%)	9 (47.4%)	0.09
Tachypnea	1 (2.9%)	1 (5.3%)	1
Dyspnea	0 (0%)	2 (10.5%)	0.12
Diarrhea	3 (8.8%)	1 (5.3%)	1
Vomiting	6 (17.6%)	4 (21.1%)	1
Abdominal pain	5 (14.7%)	1 (5.3%)	0.4
Convulsion	0 (0%)	6 (31.6%)	< 0.05
Limb pain	1 (2.9%)	2 (10.5%)	0.29
Laboratory examination			
WBC (× 10^9^/L)	6.58 (4.06, 8.61)	8.13 (5.97, 13.69)	0.02
N%	60.45 (40.98, 69.00)	71.80 (57.90, 79.00)	0.01
L%	30.50 (19.70, 49.58)	18.60 (12.70, 31.30)	0.02
M%	8.94 ± 3.49	7.53 ± 2.98	0.14
CRP (mg/L)	2.01 (1.03, 5.90)	1.93 (0.62, 15.80)	0.87
PCT (ng/mL)	0.20 (0.10, 0.39)	0.29 (0.09, 0.93)	0.34
SAA (mg/L)	49.33 (12.58, 97.09)	39.83 (15.80, 118.62)	0.80
ALT (U/L)	18.90 (13.60, 23.48)	13.70 (10.90, 1 9.60)	0.05
AST (U/L)	42.15 (31.03, 58.40)	38.00 (24.20, 53.90)	0.31
Albumin (g/L)	44.85 (42.13, 46.55)	45.50 (43.00, 47.80)	0.29
Creatinine (µmol/L)	27.25 (19.95, 31.83)	29.50 (22.60, 34.20)	0.52
Urea (mmol/L)	3.60 ± 0.88	3.94 ± 1.06	0.22
hs‐TNT (ng/mL)	0.004 (0.003, 0.005)	0.003 (0.003, 0.004)	0.09
Combined with other virus infections			
Mycoplasma	1 (2.9%)	1 (5.3%)	1
Respiratory syncytial virus	5 (14.7%)	2 (10.5%)	1
Adenovirus	4 (11.8%)	1 (5.3%)	0.64
Influenza B	2 (5.9%)	2 (10.5%)	0.61
Combined with bacterial infection	0 (0%)	7 (36.8%)	< 0.05

^a^The maximum body temperature during the course of the disease.

### 3.3. Compared With the Mild Group, the Severe Group Had a Higher Serum Level of Chemokine IP‐10

The cytokines IL‐6 and TNF‐α showed no significant difference between the mild and severe groups (*P* > 0.05), but the level of chemokine IP‐10 was statistically different between the two groups, and the severe group was higher than the mild group (*P* < 0.05, Table 3).

**Table 3:** Comparisons of cytokines between the mild and severe groups in post‐COVID‐19 influenza A lower respiratory tract infection.

Variables	Mild (*n* = 34)	Severe (*n* = 19)	*p* value
TNF‐α^a^ [*x* ± s]	33.68 ± 16.92	31.43 ± 15.81	0.64
IP‐10^b^ [M (P25, P75)]	48.85 (35.26, 62.03)	60.32 (46.39, 74.59)	0.04
IL‐6^c^ [M (P25, P75)]	13.93 (11.25, 18.84)	16.48 (13.18, 24.22)	0.14

^a^Tumor necrosis factor‐α.

^b^Interferon‐inducible protein‐10.

^c^Interleukin‐6 (IL‐6).

### 3.4. IP‐10 and WBC Serve as Diagnostic Biomarkers for Severe Infection, and Their Combined Use Demonstrates Enhanced Diagnostic Performance

The binary logistic regression analysis was performed for statistically significant markers in the univariate analysis, including WBC, N%, L%, and IP‐10, using the “enter” method. Studies have shown that WBC and IP‐10 are risk factors for developing severe influenza A LRTI after COVID‐19 infection (Table 4).

**Table 4:** The binary logistic regression analysis and ROC analysis for predicting severe post‐COVID‐19 influenza A lower respiratory tract infection.

Variables	OR	95% CI	*p* value
IP‐10	1.053	1.008–1.009	0.03
WBC	1.256	1.020–1.547	0.02

*Note:* Using the method of “enter.”

**Figure 2:**
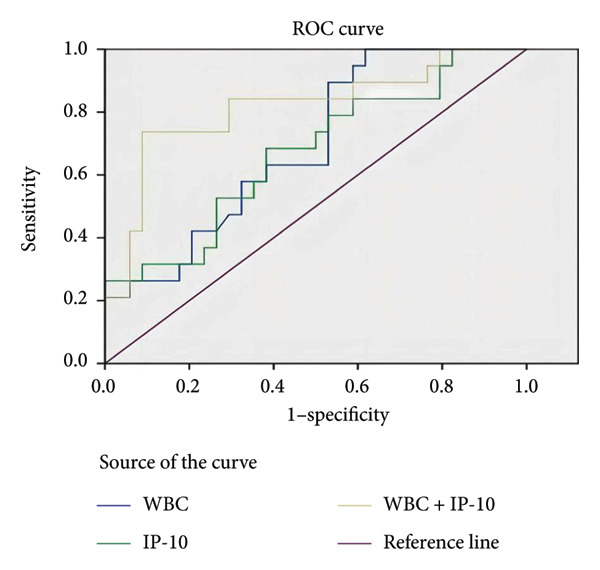
Performance of ROC curves for predicting severe post‐COVID‐19 influenza A lower respiratory tract infection.

To better explore the prediction accuracy of IP‐10, WBC, and the combination of IP‐10 and WBC for severe influenza A LRTI after COVID‐19 infection, we performed ROC analysis (Figure 2). Through ROC curve analysis, we concluded that the AUC for IP‐10 of 0.670 (95% confidence interval [CI]: 0.518–0.823; *p* < 0.05) and optimum cut‐off expression value was 54.5 pg/mL with a sensitivity of 68% and specificity of 62%; the AUC of WBC was 0.694 (95% CI: 0.552–0.836; *p* < 0.05), and at a cutoff value of 5.1 × 10^9^/L, WBC provided sensitivity and specificity of 100% and 38%, respectively. Combinations of the two indicators, IP‐10 and WBC, showed the highest AUC of 0.816 (95% confidence interval [CI]: 0.687–0.945; *p* < 0.01) (Table 5).

**Table 5:** Results of ROC analysis of IP‐10, WBC, and the combination of IP‐10 and WBC for predicting severe post‐COVID‐19 influenza A lower respiratory tract infection.

Indicators	AUC	95% CI	*p* value
IP‐10	0.670	0.518–0.823	0.04
WBC	0.694	0.552–0.836	0.02
IP‐10 + WBC	0.816	0.687–0.945	< 0.01

## 4. Discussion

Following the adjustment of China’s “dynamic zero‐COVID” policy in December 2022, a significant surge in SARS‐CoV‐2 infections was observed. Concurrently, an outbreak of influenza A virus infections occurred among children between March and April 2023. Although research on the pathogenesis, clinical manifestations, and disease progression of influenza A virus has become increasingly comprehensive, studies focusing on influenza A infection during the postpandemic transition period—particularly following widespread SARS‐CoV‐2 transmission—remain limited. The interplay between prior SARS‐CoV‐2 infection and subsequent influenza A susceptibility warrants further investigation. During the peak of the pandemic (November 2019 to November 2022), stringent NPIs were implemented across China, including enhanced hand hygiene, mandatory mask‐wearing in public settings, and social distancing. Numerous domestic and international studies have demonstrated that these behavioral and policy changes significantly disrupted the circulation and evolution of respiratory pathogens, including influenza A virus, resulting in markedly reduced detection rates [20–22]. However, following the relaxation of the “dynamic zero‐COVID” policy in late 2022, the resurgence of SARS‐CoV‐2 coincided with a rapid increase in pediatric influenza A cases, potentially attributable to accumulated immune debt due to prolonged NPIs. Research during this transitional phase has primarily focused on the epidemiological characteristics of the re‐emergence of influenza A and other pathogens after the dominance of the Omicron variant; shifts in clinical and laboratory features of viral infections before, during, and after the pandemic era; and comparative analyses of clinical presentations between children infected with SARS‐CoV‐2 versus influenza A virus [23–26]. The pandemic‐related interventions have notably altered the global transmission dynamics of seasonal influenza. Evidence indicates that 3 years after the onset of the pandemic, with the recovery of international air travel, interregional influenza lineage transmission intensity had largely returned to prepandemic levels—except for the B/Victoria lineage—highlighting the resilience of global influenza dissemination patterns despite major disruptions [23]. Additionally, an epidemiological study conducted in Shijiazhuang, China, comparing acute respiratory infection patients during the pandemic (January 2021–December 2022) and after restriction easing (January 2022–December 2023) revealed increased detection rates of non‐SARS‐CoV‐2 pathogens in 2023, with influenza A virus being the most prevalent at 11.35%, accompanied by alterations in its typical seasonal pattern [24]. A retrospective analysis of 1063 hospitalized children from two tertiary hospitals in Guangdong Province identified distinct clinical and laboratory profiles between those infected with SARS‐CoV‐2 and those with influenza A virus [26]. Nevertheless, data characterizing children who develop influenza A infection following prior SARS‐CoV‐2 exposure during this postrestriction period remain scarce.

To investigate the clinical features, laboratory parameters, and cytokine profiles in children with influenza A‐associated LRTI following previous SARS‐CoV‐2 infection, we enrolled 53 hospitalized pediatric patients who had documented SARS‐CoV‐2 infection between November 2022 and February 2023 and were subsequently diagnosed with influenza A LRTI. Patients were stratified into mild and severe groups based on clinical presentation for comparative analysis. Prior to analysis, we compared the prevalence of prior SARS‐CoV‐2 infection between the two groups and found a higher proportion in the severe group, suggesting an increased risk of severe influenza A outcomes following prior coronavirus infection. Compared to prepandemic influenza seasons, when peak activity typically occurred between January and February [27], the current wave peaked later, from March to April 2023—a shift potentially linked to the resumption of population mobility and normal social activities following the relaxation of control measures in early 2023 [28, 29]. Consistent with existing literature indicating heightened vulnerability to acute pneumonia in children under 5 years of age [18, 30], our cohort consisted exclusively of LRTI cases, predominantly aged 3–5 years. Clinically, while respiratory symptoms such as cough and dyspnea were predominant in prepandemic influenza cases, neurological manifestations—including seizures and altered consciousness—emerged as prominent features during the pandemic period (2019–2022). Notably, one study highlighted that during the COVID‐19 pandemic, acute necrotizing encephalopathy was associated with an increased risk of severe outcomes and influenza‐related mortality [19]. In our study, although influenza‐associated acute necrotizing encephalopathy was not definitively diagnosed in the severe group, consistent with previous findings, convulsions emerged as a predominant clinical manifestation alongside common respiratory symptoms such as fever and cough. Therefore, during influenza seasons, pediatricians should remain vigilant not only to the progression of respiratory symptoms in children but also to neurological manifestations, including seizures, lethargy, and altered consciousness. Laboratory findings revealed that the severe group exhibited higher white blood cell (WBC) counts and neutrophil percentages, along with lower lymphocyte percentages, compared to the mild group. In a study on the prediction of disease severity in influenza B pneumonia, it was noted that lymphopenia is an independent risk factor for severe disease progression [31]. Elevated neutrophilic indices may reflect secondary bacterial co‐infections, which are commonly associated with severe influenza. Previous studies report viral‐bacterial co‐infection rates of 30%–50% in community‐acquired pneumonia (CAP) cases, including those involving influenza, and such co‐infections are linked to greater disease severity and mortality [32–35]. Increased susceptibility may stem from perturbations in pulmonary immunity and early depletion of alveolar macrophages—cells critical for pathogen clearance and immune activation [36]. Notably, lymphopenia observed in the severe group aligns with prior reports identifying peripheral lymphocyte reduction as an early immunological hallmark of influenza A infection [37, 38]. Comparative studies indicate that children with SARS‐CoV‐2 infection are typically younger (0–3 years), more likely to present with anorexia and respiratory distress, and uniquely susceptible to multisystem inflammatory syndrome in children (MIS‐C), particularly following Omicron infection. Laboratory markers show higher CRP elevation in SARS‐CoV‐2 cases, whereas lymphopenia is more characteristic of influenza A infection [26]. Thus, the clinical and laboratory profiles of children in our cohort—those with influenza A following prior SARS‐CoV‐2 infection—more closely resemble those reported for influenza during the pandemic era than either prepandemic influenza or primary SARS‐CoV‐2 infection.

Both the influenza A virus and SARS‐CoV‐2 are respiratory pathogens that utilize specific host receptors for cellular entry, initiating disease pathogenesis. In pediatric populations, influenza A is associated with higher morbidity and mortality and often presents with atypical symptoms. In contrast, SARS‐CoV‐2 infections in children are generally less frequent and milder in severity [39, 40]. Despite differences in transmissibility and virulence, cytokines play a pivotal role in mediating immunopathology and determining disease severity in both infections. These signaling molecules regulate the balance between cellular and humoral immunity; however, excessive production of proinflammatory cytokines such as IL‐6 and TNF‐α can trigger uncontrolled immune activation, leading to widespread tissue damage—a phenomenon termed “cytokine storm.” In this study, serum levels of TNF‐α, IP‐10, and IL‐6 were measured in both mild and severe patient groups. No significant association was observed between disease severity in post‐SARS‐CoV‐2 influenza A virus‐induced LRTI and the levels of IL‐6 or TNF‐α. However, IP‐10 (CXCL10) levels exhibited a statistically significant difference between the two groups, with higher concentrations detected in the severe group compared to the mild group. IP‐10 is primarily secreted by neutrophils, dendritic cells, astrocytes, fibroblasts, endothelial cells, and hepatocytes, and functions as a potent chemokine. Upon binding to its receptor CXCR3, it activates T cells, NK cells, and monocytes, promoting their recruitment to sites of inflammation [41]. Consequently, the IP‐10–CXCR3 axis may perpetuate inflammatory responses, exacerbate tissue injury, and amplify neutrophil‐mediated lung damage during viral infections [42], positioning it as a potential biomarker for assessing disease progression and severity in viral respiratory illnesses. Findings from a multicenter cytokine profiling study in Egyptian children and adolescents similarly identified elevated serum IP‐10 as an independent predictor of severe COVID‐19 pneumonia, supporting our results [16]. Binary logistic regression analysis of variables identified through univariate testing confirmed that both IP‐10 level and WBC count were independent risk factors for severe influenza A‐related LRTI following SARS‐CoV‐2 infection. While ROC curve analysis indicated limited diagnostic accuracy when each marker was used alone, their combination significantly improved predictive performance.

In our cohort, a higher proportion of children with severe influenza A LRTI had prior SARS‐CoV‐2 infection, suggesting increased susceptibility to severe outcomes. However, clinical manifestations were relatively less severe compared to historical H1N1 cohorts, with fewer cases progressing to ARDS or multiorgan failure. This may be partially attributed to sample size limitations, but we cannot exclude potential immunomodulatory effects of prior SARS‐CoV‐2 infection, including transient immune training, which merits further investigation.

This study has several limitations. First, it is a single‐center, retrospective analysis with a relatively small sample size, which may introduce selection bias. Second, longitudinal monitoring of clinical and immunological parameters throughout the entire disease course was not fully captured. Finally, the underlying mechanisms by which prior SARS‐CoV‐2 infection influences subsequent influenza A pathogenesis require deeper mechanistic exploration.

## 5. Conclusions

Our study shows that compared with previous influenza A infections, severe LRTI of influenza A virus is more likely to occur after COVID‐19 infection, but the symptoms in severe children are relatively mild, and the rate of convulsions and secondary bacterial infection is increased. In the study of the laboratory tests and the levels of cytokines in the mild and severe groups of children, it was found that the combination of WBC and IP‐10 had an important value in the diagnosis of the severity of the disease.

Finally, through our study, we found the changes in the clinical characteristics and levels of inflammatory cytokines of H1N1 infection before and after the novel coronavirus, which lays the foundation for a better understanding of the mechanism of influenza A or other virus infection after novel coronavirus infection and provides a diagnostic basis for clinical pediatricians to detect severe children in early stages.

## Ethics Statement

This study was reviewed and approved by the Ethics Committee of the Affiliated Hospital of Southwest Medical University (Ethics Review Approval Number: KY202337).

## Disclosure

All authors approved the submitted version [43].

## Conflicts of Interest

The authors declare no conflicts of interest.

## Author Contributions

Y.Q., Y.Z., and C.L. designed the experiments. X.L., H.D., and Y.Q. collected samples. X.X., Q.Z., and M.H. analyzed the data. Y.Q. and L.X. writing–original draft. C.L. and Y.Z. writing–review and editing. C.L. and Y.Z. project administration. All authors contributed to the article. Y.Q. and X.X. contributed equally to this work and share first authorship.

## Funding

This work was supported by a doctoral Research Initiation Fund of Affiliated Hospital of Southwest Medical University (20082).

## Data Availability

The data that support the findings of this study are available from the corresponding author upon reasonable request.
